# Profiling Hydrophilic *Cucurbita pepo* Seed Extracts: A Study of European Cultivar Variability

**DOI:** 10.3390/plants14152308

**Published:** 2025-07-26

**Authors:** Adina-Elena Grasu, Roman Senn, Christiane Halbsguth, Alexander Schenk, Veronika Butterweck, Anca Miron

**Affiliations:** 1 Department of Pharmacognosy-Phytotherapy, Faculty of Pharmacy, Grigore T. Popa University of Medicine and Pharmacy, Universitatii Str. 16, 700115 Iasi, Romania; adina-elena.grasu@d.umfiasi.ro; 2 Max Zeller & Söhne AG, Seeblickstrasse 4, 8590 Romanshorn, Switzerland; roman.senn@zellerag.ch (R.S.); christiane.halbsguth@zellerag.ch (C.H.); aschenk58@icloud.com (A.S.)

**Keywords:** *Cucurbita pepo*, lignans, cucurbitin, trigonelline, raffinose, stachyose, verbascose

## Abstract

*Cucurbita pepo* (CP) seeds are traditionally used to alleviate lower urinary tract symptoms associated with benign prostatic hyperplasia and overactive bladder. While these effects are often attributed to lipophilic constituents, recent studies have highlighted the therapeutic potential of oil-free hydroethanolic extracts. However, their composition remains insufficiently characterized, considering the species’ significant phenotypic and phytochemical variability. This study aimed to characterize the phytochemical profile of hydrophilic hydroethanolic seed extracts from ten CP cultivars originating from different European regions, with a focus on compositional variability. The elemental composition, along with primary and secondary metabolites, was analyzed using established spectroscopic and chromatographic methods. The extracts showed considerable variation in protein (45.39 to 114.58 mg/g dw) and free amino acid content (46.51 to 111.10 mg/g dw), as well as differences in elemental composition. Principal component analysis revealed distinct clustering patterns, with several samples displaying metabolite profiles comparable to the *Cucurbita pepo* var. *styriaca* variety currently recommended by the European Pharmacopoeia (Ph. Eur.) and the Committee on Herbal Medicinal Products (HMPC). These findings open the possibility of using other CP varieties as alternative sources for extract preparation and offer novel insights into the composition of less explored hydrophilic extracts derived from CP seeds.

## 1. Introduction

*Cucurbita pepo* L. (CP), a versatile member of the Cucurbitaceae family, originating from Mexico [1], is known for its wide range of morphological varieties including pumpkins, squashes, and zucchini [2]. The plant is cultivated globally for culinary applications of both fruits and seeds [3], but it has gathered significant interest over the years, particularly for its therapeutic potential [4]. Pumpkin flesh and seed extracts have been investigated for their antidiabetic properties both in vitro and in vivo, demonstrating alpha-amylase and alpha-glucosidase inhibitory activities [5] and showing dose-dependent hypoglycemic effects when administered orally to diabetic rats [6]. Furthermore, pumpkin seeds and their extracts, whether whole extracts obtained from seeds [7], or cold pressed oil [8,9], particularly alleviated overactive bladder (OAB) [10] and benign prostatic hyperplasia (BPH) symptoms [9].

In recent years, aqueous extracts from CP seeds have gathered attention for their bioactivity, particularly in alleviating symptoms of OAB [11,12], a condition that affects both men and women globally. OAB is characterized by sudden, uncontrollable urges to urinate, often accompanied by increased frequency and nocturia, significantly impairing quality of life [13]. A randomized, double-blind, placebo-controlled trial demonstrated that a formulation combining oil-free pumpkin seed extract with soy germ extract significantly reduced urinary frequency, urgency, and incontinence in women with OAB over 12 weeks [14]. Another study showed that a similar product effectively improved symptoms of stress urinary incontinence in women over a six-week period [15]. Additionally, a three-month pilot study found that oil-free pumpkin seed extract, used as a monotherapy, improved lower urinary tract symptoms (LUTSs) in men with BPH without notable side effects [16]. These clinical findings suggest that constituents beyond phytosterols [17] and fatty acids [18] may contribute to the therapeutic effects observed in LUTSs.

In 2013, the Committee on Herbal Medicinal Products (HMPC) published a monograph recognizing the traditional use of CP seeds for the relief of LUTSs associated with BPH or OAB, based on long-standing medicinal use [19]. This monograph refers to the herbal substance as “whole, ripe, and dried seeds”, without specifying a particular variety. However, the accompanying assessment report indicates that CP convar. *citrullina* var. *styriaca* is typically used for pharmaceutical purposes [20]. Commonly known as the “Styrian oil pumpkin”, this variety arose in the 19th century from a natural mutation of a single recessive gene [21], resulting in seeds with a very thin outer layer that facilitated cold-pressed oil production [22]. Although a thin membrane remains, these seeds are generally referred to as “hull-less” or “naked” due to the absence of a lignified hull [23]. As of July 2023, the European Pharmacopoeia (Ph. Eur.) introduced a monograph on pumpkin seeds [24], defining the plant material as “whole, dried, ripe seeds of hull-less seeded varieties of *Cucurbita pepo* L.”, echoing the HMPC guidance but without restricting it to a specific cultivar [20].

CP exhibits substantial variability across its cultivars [25,26,27], both in morphological traits and chemical composition [28]. Numerous studies have reported differences in fatty acid profiles, phytosterol content, and other bioactive compounds, largely influenced by genetic diversity and environmental conditions [29,30]. This inherent variability poses a challenge to the reproducibility of hydrophilic extract compositions and the consistency of their associated biological activities across different CP varieties.

Although oil-free pumpkin seed extracts exhibited clinical benefits in managing LUTSs, pharmacopeial standards continue to focus primarily on lipophilic constituents such as fatty acids and phytosterols. In contrast, the hydrophilic fraction remains poorly characterized and is largely overlooked in standardization frameworks. This study aimed to comprehensively investigate the chemical composition of hydrophilic extracts from CP seeds, comparing two hull-less varieties with eight hulled ones. The objectives were to identify key components that could serve as chemical markers for standardizing hydrophilic extracts intended for medicinal use, to highlight major compositional differences between varieties and their implications for quality control, and to assess whether the *styriaca* variety possesses a distinct profile that justifies its preferential inclusion in official monographs. To accomplish our goal, the extracts were characterized starting with elemental analysis to understand the sample matrix. Broad profiling of hydrophilic constituents was achieved using high performance thin layer chromatography (HPTLC) and spectrophotometric methods. Subsequently, targeted analyses were conducted using ultra performance liquid chromatography (UPLC) coupled with a photodiode array detector (PDA), single quadrupole mass detector (QDA), high resolution mass spectrometry (HRMS), or evaporative light scattering detector (ELSD) to identify and quantify primary and secondary metabolites.

## 2. Results

### 2.1. Water Content and Elemental Analysis

#### 2.1.1. Water Content

The water content of the extracts was determined prior to this study using Karl Fischer titration method and incorporated into data normalization. The results are presented for each sample in Table S1 (Supplementary Materials). Accordingly, all further results were expressed as mg/g of extract dry weight (dw), enabling direct comparison across samples, with the CP convar. *citrullina* var. *styriaca* (SK) extract used as a reference. The remaining nine extracts were coded as follows: SKR (CP var. *styriaca* cultivar Gleisdorfer Rustikal), LN (CP—Lady Nail), SW (CP—Snow White), RZ (CP var. *giromontia*—Radu), SS (CP—Shine Skin), GA (CP—Greek Cultivar), GV (CP—Grey Volga), BBZ (CP var. *cylindrica*—Black Beauty), and HV (CP—Hungarian Cultivar).

#### 2.1.2. Metal Analysis

**Figure 1:**
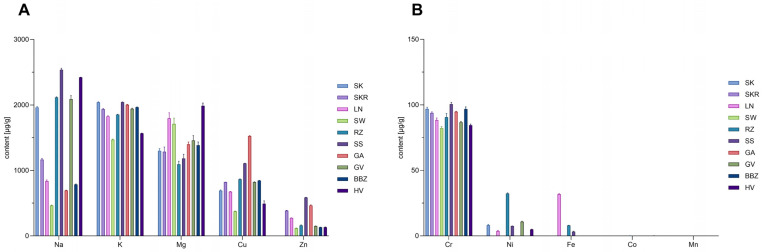
Bar chart of mean elemental concentrations (µg/g dw) in *Cucurbita pepo* (CP) seed hydrophilic extracts, expressed as mean ± SD (*n* = 3 technical replicates). (**A**) Higher-abundance elements (Na, K, Mg, Cu > 500 µg/g dw in at least one extract). (**B**) Lower-abundance elements (Zn, Cr, Ni, Fe, Co, Mn). Grouping was based on concentration range allowing appropriate scaling and clearer visualization of differences across samples. Sample codifications are as follows: SK (CP convar. *citrullina* var. *styriaca*), SKR (CP var. *styriaca* cultivar Gleisdorfer Rustikal), LN (CP—Lady Nail), SW (CP—Snow White), RZ (CP var. *giromontia*—Radu), SS (CP—Shine Skin), GA (CP—Greek Cultivar), GV (CP—Grey Volga), BBZ (CP var. *cylindrica*—Black Beauty), HV (CP—Hungarian Cultivar).

Quantitative flame atomic absorption spectroscopy (FAAS) analysis revealed variations in elemental composition among hydrophilic extracts, as depicted in Figure 1A,B. Regarding the macroelements, relative to the SK sample (1962.92 µg/g dw), Na concentration was elevated in samples SS (2538.71 ± 22.85 µg/g dw) and HV (2419.78 ± 4.84 µg/g dw), in contrast with the lowest level in SW (464.76 ± 6.51 µg/g dw). K was present overall in high concentrations, with the SK sample (2041.56 ± 8.17 µg/g dw) being the most abundant, while the SW sample (1468.59 ± 10.28 µg/g dw) presented the lowest concentration. Mg levels also showed variation across samples, peaking in the HV sample (1988.81 ± 41.76 µg/g dw), as opposed to the RZ sample (1092.67 ± 48.08 µg/g dw).

The GA sample, compared to the SK control sample, had the highest level of Cu (1525.40 ± 6.10 vs. 692.50 ± 10.45 µg/g dw). Zn was quantified only in trace amounts in the SK sample (0.9 ± 0.00 µg/g dw), while markedly higher levels were observed in all the other samples, varying between SW and SS (117.31 ± 3.75 and 587.60 ± 4.08 µg/g dw). Cr displayed consistent distributions across varieties with the SK sample containing 96.94 ± 1.36 µg/g dw. Ni displayed variable accumulation in six (SK, LN, RZ, SS, GV, HV) of the ten tested extracts. Fe was sporadically detected in the SS (3.180 ± 0.38 µg/g dw), RZ (0.820 ± 0.08 µg/g dw) and LN samples (32.00 ± 0.38 µg/g dw), while Co and Mn levels were below the limit of quantification (LOQ) in all samples.

#### 2.1.3. Total Carbon and Total Nitrogen Content

Analysis of the carbon and nitrogen contents of the extracts revealed variations among samples, as shown in Table 1. Notably, the GV sample exhibited higher levels of total carbon (TC) compared with the SK control sample (531.27 ± 0.74 vs. 452.32 ± 1.04 mg/g dw), while TC contents in GA and BBZ samples were lower (308.74 ± 0.40 and 304.38 ± 1.95 mg/g dw, respectively). Inorganic carbon (IC) level in the SK sample differed from that in the BBZ sample, which had the highest level (2.95 ± 0.17 vs. 7.05 ± 0.13 mg/g dw) among the tested extracts. Total nitrogen content (TN) in the SK sample was lower than those in SW and HV samples (14.32 ± 0.02 vs. 26.57 ± 0.03 and 27.09 ± 0.24 mg/g dw, respectively), while the differences between the control sample and the remaining samples were less pronounced.

### 2.2. LC-PDA-HRMS Analysis

Phytochemical screening of CP hydrophilic extracts began with HPTLC, revealing the presence of mono and oligosaccharides as well as amino acids, while phenolic acids and flavonoids were not detected (Figures S1–S3). UPLC-PDA-HRMS analysis confirmed a diverse phytochemical profile (Table A1). Cucurbitin, trigonelline, and nicotinic acid were consistently identified across all extracts, along with nucleosides such as adenosine and guanosine; inosine was absent. Phenolic glycosides (cucurbitosides A-M, except G) and gibberellins (A1, A8, A39) were uniformly present. Most flavonoids including epigallocatechin, catechin, quercetin derivatives, and kaempferol were not detected, while some samples only showed trace amounts of rutin (SK, RZ, SS, and GA), orientin, and homoorientin (SW, RZ, SS, GA, and BBZ). Chlorogenic acid was variably present in SK, LN, RZ, GA, and BBZ, while gallic and protocatechuic acids were undetected. Lignans such as secoisolariciresinol (Seco), lariciresinol (Lar), and pinoresinol (Pino) were found in several samples (LN, RZ, SS, GA, GV, BBZ, HV) but were absent in SK and SKR, suggesting seed coat localization. Cucurbitacins (A, D, E, I) were not detected, with only trace cucurbitacin B observed in LN.

### 2.3. Analysis of Carbohydrates

**Figure 2:**
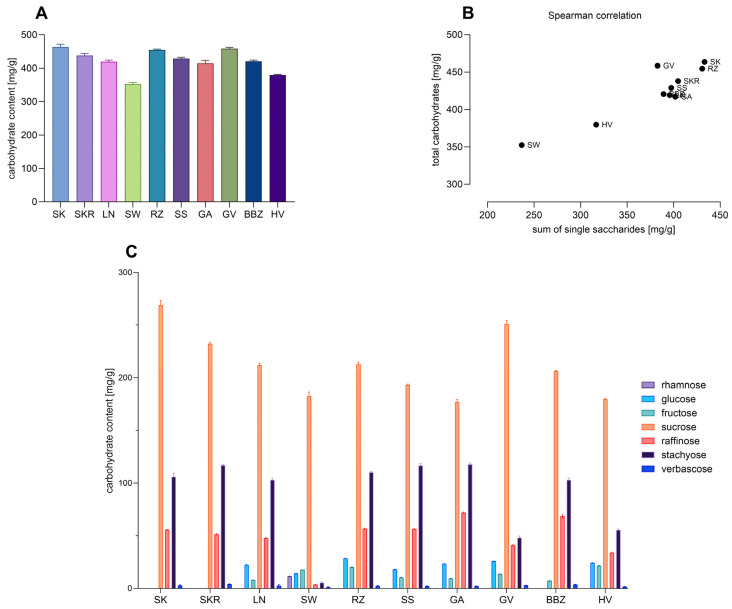
(**A**) Total carbohydrate content in CP seed hydrophilic extracts expressed as mean ± SD (*n* = 3 technical replicates). (**B**) Dot plot revealing the Spearman correlation between the sum of quantified saccharides and the total carbohydrate content. (**C**) Bar chart showing the content of individually quantified saccharides in *Cucurbita pepo* (CP) seed hydrophilic extracts, expressed as mean ± SD (*n* = 3 technical replicates). Sample codifications are as follows: SK (CP convar. *citrullina* var. *styriaca*), SKR (CP var. *styriaca* cultivar Gleisdorfer Rustikal), LN (CP—Lady Nail), SW (CP—Snow White), RZ (CP var. *giromontia*—Radu), SS (CP—Shine Skin), GA (CP—Greek Cultivar), GV (CP—Grey Volga), BBZ (CP var. *cylindrica*—Black Beauty), HV (CP—Hungarian Cultivar).

Carbohydrate composition was assessed via the phenol–sulfuric acid method and UPLC-ELSD, as shown in Figure 2A,C. Total carbohydrate content was highest in the SK sample (463.62 ± 8.18 mg/g dw) and lowest in the SW sample (352.41 ± 4.85 mg/g dw); SKR, RZ, and GV showed similar values to the SK sample. Sucrose, (Figure 2C) dominated across all samples, with the highest level in SK (268.63 ± 4.87 mg/g dw) and the lowest in SW (182.74 ± 3.73 mg/g dw). Stachyose varied widely, from 5.48 ± 0.82 mg/g dw (SW) to 117.00 ± 0.94 mg/g dw (SKR), while raffinose was notably high in GA and BBZ (72.10 ± 0.65 and 68.76 ± 1.56 mg/g dw).

Verbascose was present in minor but consistent amounts across all samples (1.32–3.93 mg/g dw). Glucose and fructose were mainly found in RZ, HV, and GV samples, but were absent or present in trace amounts in SK and SKR. A moderate positive correlation (Spearman ρ = 0.6606, *p* < 0.0438) was observed between total carbohydrate content and the sum of quantified saccharides (Figure 2B), supporting the consistency and representativeness of the analytical methods used.

### 2.4. Analysis of Proteins and Free Amino Acids

**Figure 3:**
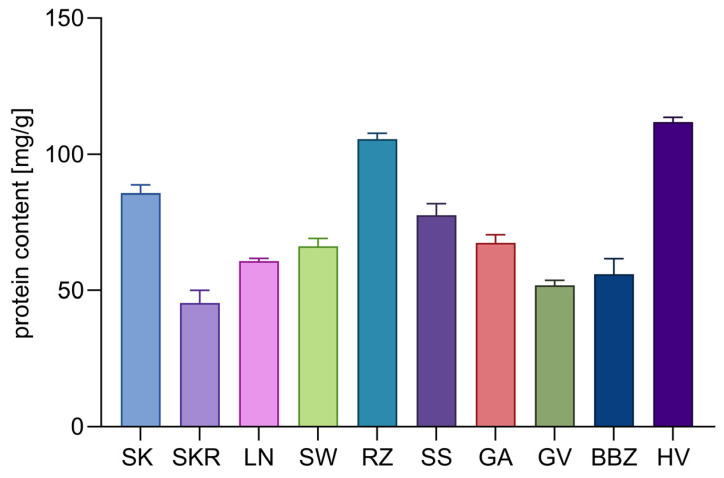
Bar chart of mean protein contents (mg/g dw) in *Cucurbita pepo* (CP) seed hydrophilic extracts, expressed as mean ± SD (*n* = 3 technical replicates). Sample codifications are as follows: SK (CP convar. *citrullina* var. *styriaca*), SKR (CP var. *styriaca* cultivar Gleisdorfer Rustikal), LN (CP—Lady Nail), SW (CP—Snow White), RZ (CP var. *giromontia*—Radu), SS (CP—Shine Skin), GA (CP—Greek Cultivar), GV (CP—Grey Volga), BBZ (CP var. *cylindrica*—Black Beauty), HV (CP—Hungarian Cultivar).

Total protein content, as determined by spectrophotometric analysis, is presented in Figure 3. The highest levels were observed in HV and RZ samples (111.83 ± 1.80 and 105.54 ± 2.16 mg/g dw, respectively), while SKR, GV, and SW showed the lowest contents (45.39–66.19 mg/g dw).

**Figure 4:**
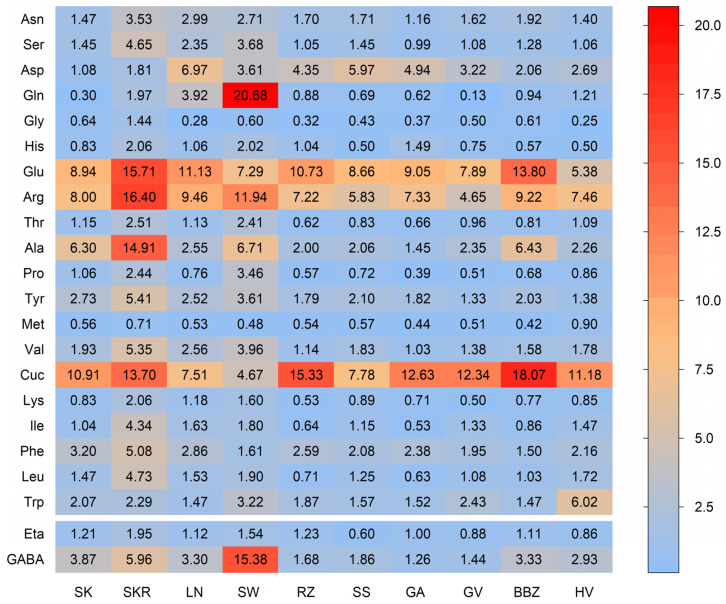
Heat map illustrating the concentrations of free amino acids (mg/g dw) in *Cucurbita pepo* (CP) seed hydrophilic extracts, expressed as mean (*n* = 3 technical replicates). The color gradient reflects the variation in concentration across the samples, with red indicating higher concentrations and blue indicating lower concentrations. Analyzed amino acids include the following: asparagine (Asn), serine (Ser), aspartic acid (Asp), glutamine (Gln), glycine (Gly), histidine (His), glutamic acid (Glu), arginine (Arg), threonine (Thr), alanine (Ala), proline (Pro), tyrosine (Tyr), methionine (Met), valine (Val), cucurbitin (Cuc), lysine (Lys), isoleucine (Ile), phenylalanine (Phe), leucine (Leu), tryptophan (Trp), ethanolamine (Eta), and gamma-aminobutyric acid (GABA). Sample codifications are as follows: SK (CP convar. *citrullina* var. *styriaca*), SKR (CP var. *styriaca* cultivar Gleisdorfer Rustikal), LN (CP—Lady Nail), SW (CP—Snow White), RZ (CP var. *giromontia*—Radu), SS (CP—Shine Skin), GA (CP—Greek Cultivar), GV (CP—Grey Volga), BBZ (CP var. *cylindrica*—Black Beauty), HV (CP—Hungarian Cultivar).

To support qualitative assessment, a rapid UPLC-UV method was developed for the simultaneous identification and quantification of twenty-three aminic compounds, including twenty proteinogenic amino acids, one non-proteinogenic amino acid (cucurbitin), and two amines (ethanolamine and GABA). Their distribution is presented as a heat map in Figure 4.

Glu, Arg, and Ala were the most abundant proteinogenic amino acids, while Met, Gly, and Cys (undetectable) were consistently low across all extracts. SKR showed a higher total aminic content than SK (119.01 ± 0.40 vs. 61.07 ± 0.28 mg/g dw). SW had notably high Gln and GABA levels (20.68 ± 1.88 and 15.38 ± 0.38 mg/g dw). Cuc was most concentrated in BBZ and RZ (18.07 ± 0.35 and 15.33 ± 1.44 mg/g dw), followed by SKR, GA, GV, and HV. Gly, Met, and His remained low across all samples. Tyr peaked in SKR (5.41 ± 0.54 mg/g dw), and Trp peaked in HV (6.02 ± 0.23 mg/g dw), exceeding levels in the SK control. These results highlight notable variation in nitrogen-containing compound profiles among the extracts.

### 2.5. Analysis of Lignans

**Figure 5:**
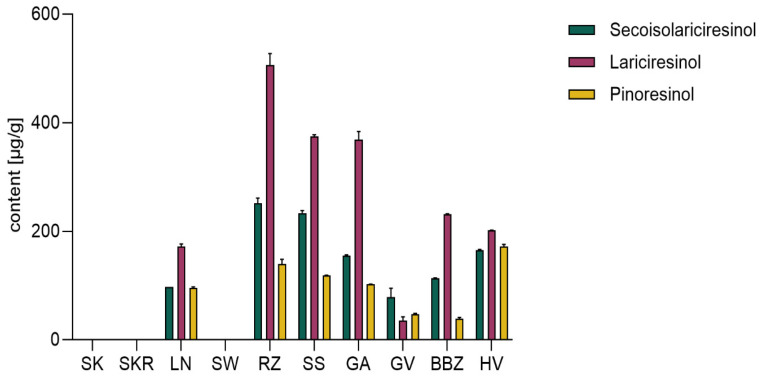
Lignans quantification in hydrophilic extracts of *Cucurbita pepo* (CP) seeds, expressed as mean ± SD (*n* = 3 technical replicates). Sample codifications are as follows: SK (CP convar. *citrullina* var. *styriaca*), SKR (CP var. *styriaca* cultivar Gleisdorfer Rustikal), LN (CP—Lady Nail), SW (CP—Snow White), RZ (CP var. *giromontia*—Radu), SS (CP—Shine Skin), GA (CP—Greek Cultivar), GV (CP—Grey Volga), BBZ (CP var. *cylindrica*—Black Beauty), HV (CP—Hungarian Cultivar).

Analysis of lignans revealed pronounced differences between the extracts, particularly in Lar levels, which peaked in RZ (506.27 ± 21.11 µg/g dw), followed by SS (374.95 ± 2.79 µg/g dw) and GA (368.38 ± 15.42 µg/g dw). Seco showed a similar distribution pattern, while Pino was more evenly distributed across samples, but much less concentrated. Lignans were absent in the extracts derived from hull-less varieties SK and SKR (Figure 5), while the SW sample showed only trace amounts below the LOQ.

### 2.6. Analysis of Trigonelline

The trigonelline content also varied considerably among the tested extracts (Table 2), ranging from 0.41 ± 0.01 mg/g dw in GV and BBZ samples to 3.45 ± 0.03 mg/g dw in SW. The highest concentration was determined in the SW extract, followed by the SS, LN, and SK samples (1.97 ± 0.00, 1.76 ± 0.02, and 1.76 ± 0.00 mg/g dw). Samples extracted from zucchini varieties (BBZ and RZ) exhibited a low trigonelline content, below 0.50 mg/g dw (0.41 ± 0.01 and 0.46 ± 0.01 mg/g dw, respectively).

### 2.7. Principal Component Analysis (PCA)

**Figure 6:**
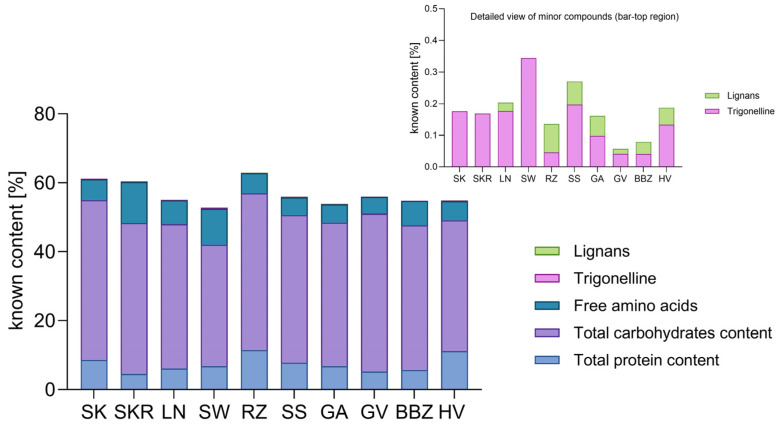
Contribution of various phytochemicals to the total mass of *Cucurbita pepo* (CP) seed hydrophilic extracts. Minor compounds below 0.5% at the bar-top region are shown as an inset. Sample codifications are as follows: SK (CP convar. citrullina var. *styriaca*), SKR (CP var. *styriaca* cultivar Gleisdorfer Rustikal), LN (CP—Lady Nail), SW (CP—Snow White), RZ (CP var. *giromontia*—Radu), SS (CP—Shine Skin), GA (CP—Greek Cultivar), GV (CP—Grey Volga), BBZ (CP var. *cylindrica*—Black Beauty), HV (CP—Hungarian Cultivar).

Prior to multivariate analysis, the contribution of quantified compounds to the total dry weight of CP seed hydrophilic extracts was assessed. As shown in Figure 6, carbohydrates consistently represented the major fraction across all samples, whereas differences in protein and free amino acids levels highlighted compositional variability among cultivars. Trigonelline and lignans, although present at relatively low levels, contributed to the further chemical differentiations. This heterogeneity in phytochemical composition supported the use of multivariate analysis in identifying compositional trends among the extracts.

**Figure 7:**
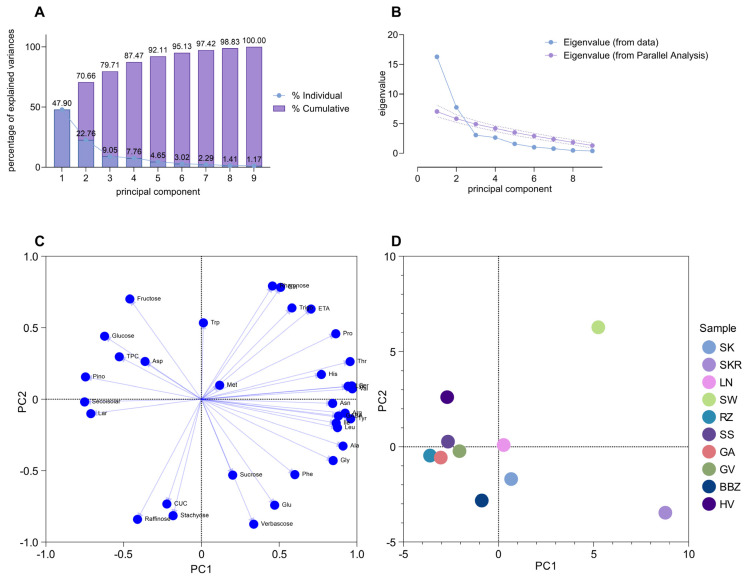
Principal component analysis (PCA) showing the variability of the phytochemical composition of *Cucurbita pepo* (CP) seed hydrophilic extracts. (**A**) Bar chart showing the percentage of explained variance for each principal component. (**B**) Eigenvalues of each PC. (**C**) Loading plot displaying the relationship between phytochemicals and the two significant PCs. (**D**) Score plot showing the samples distribution in the two-dimensional plans obtained from two significant PCs. Sample codifications are as follows: SK (CP convar. *citrullina* var. *styriaca*), SKR (CP var. *styriaca* cultivar Gleisdorfer Rustikal), LN (CP—Lady Nail), SW (CP—Snow White), RZ (CP var. *giromontia*—Radu), SS (CP—Shine Skin), GA (CP—Greek Cultivar), GV (CP—Grey Volga), BBZ (CP var. *cylindrica*—Black Beauty), HV (CP—Hungarian Cultivar).

As a methodological consideration, PCA was performed exclusively on quantitatively determined compounds, and therefore does not capture the potential contribution of trace-level constituents identified through LC-PDA-HRMS analysis. The first two principal components (PCs) accounted for 70.62% of the total variance (PC1: 46.68%, PC2: 23.94%; Figure 7A). The scree plot and parallel analysis (Figure 7B) confirmed that only PC1 and PC2 captured meaningful variation, as subsequent components showed eigenvalues below the significance threshold. Loading plots (Figure 7C) revealed that PC1 was positively associated with free amino acids (e.g., Val, Thr, Lys, Arg; loadings: 0.871 to 0.972) and negatively with lignans (Seco, Lar, Pino; loadings: −0.711 to −0.750). PC2 showed positive correlations with rhamnose, fructose, and glutamine (loadings: 0.701 to 0.792), and negative correlations with oligosaccharides (loadings: −0.841 to −0.875).

**Figure 8:**
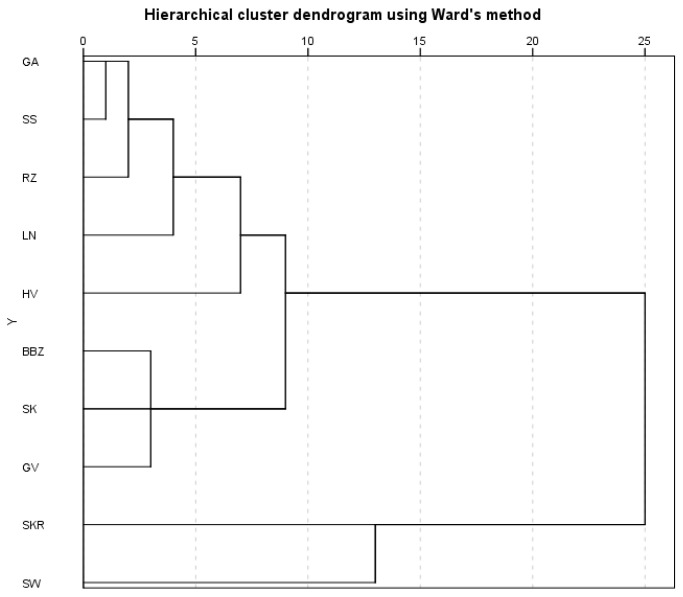
Hierarchical clustering analysis of *Cucurbita pepo *(CP) seed hydrophilic extracts based on 35 phytochemical variables. Clustering was performed using Ward’s linkage method and Euclidean distance. Two main clusters were identified, with cluster I further divided into three sub-clusters, reflecting varying degrees of metabolic similarity among the samples. Sample codifications are as follows: SK (CP convar. *citrullina* var. *styriaca*), SKR (CP var. *styriaca* cultivar Gleisdorfer Rustikal), LN (CP—Lady Nail), SW (CP—Snow White), RZ (CP var. *giromontia*—Radu), SS (CP—Shine Skin), GA (CP—Greek Cultivar), GV (CP—Grey Volga), BBZ (CP var. *cylindrica*—Black Beauty), HV (CP—Hungarian Cultivar).

The PC1 vs. PC2 biplot (Figure 7D) showed a clear separation among samples, reflecting compositional differences among the extracts. The SW and SKR samples, positioned on the right side, had distinct profiles, with the SW sample (upper right quadrant) due to high amino acids and monosaccharides, and the SKR sample (lower right quadrant) due to elevated amino acids and oligosaccharides. The SK, LN, and BBZ samples clustered near the origin, indicating similar compositions. Hierarchical cluster analysis using Ward’s linkage method based on Euclidean distance (Figure 8) supported the PCA results, revealing two main clusters. Cluster I included most samples, with the following subgroups: GA, SS, and RZ (closely related); LN and HV (moderately similar); and SK, GV, and BBZ (maintaining internal similarity). Cluster II grouped the SKR and SW samples, highlighting their distinct metabolic profiles.

## 3. Discussion

CP, a widely cultivated [28] and polymorphic species [25,26] of the Cucurbitaceae family, is traditionally used for managing LUTSs associated with BPH and OAB [19,20]. While its medicinal effects are largely attributed to lipophilic compounds like phytosterols, fatty acids, and tocopherols [8], emerging evidence suggests hydrophilic constituents such as sugars, amino acids, and polar secondary metabolites may also contribute to its pharmacological activity [14,16]. However, the hydrophilic fraction remains poorly characterized, and differences among CP varieties are understudied. As seed composition is crucial for quality control and standardization, this study provides a comprehensive comparison of hydrophilic extracts from ten CP varieties, highlighting their chemical diversity and identifying potential alternatives to CP convar. *citrullina* var. *styriaca*.

The hydrophilic extracts showed a varied mineral profile with notable differences in both macro and microelements. Due to low extraction yields (1.67–2.5%) and selective solubility, mineral contents cannot be directly extrapolated to whole seeds. Na levels ranged from 464.76 to 2538.71 µg/g dw, while K and Mg levels (1468.59–2041.56 µg/g and 1092.67–1988.81 µg/g dw, respectively) were 3–8 times lower than in seeds or oil cakes [31], aligning with their aqueous extractability. Notably, Cr (82.24–100.55 µg/g dw) and Cu (376.77–1525.40 µg/g dw) levels were higher than the literature values [30,31,32]. However, at typical intake levels of 350–875 mg/day dry extract [11,12,16], the estimated amounts of both elements remain below the permitted daily exposure limits established by the International Council for Harmonisation (ICH) guidelines, specifically, 10,700 µg/day for Cr and 3,400 µg/day for Cu [33]. Therefore, despite elevated concentrations relative to the literature data, the estimated intakes are well within internationally accepted safety thresholds and do not pose a toxicological concern according to ICH guideline and Codex nutrient reference values [33,34].

Zn levels in the extracts ranged from 0.90 ± 0.00 to 587.60 ± 4.08 µg/g dw, suggesting wide variability that may reflect differences in extraction efficiency and cultivar genetics. Despite some higher values, all Zn concentrations remained below the adult recommended daily intake (8–11 mg/day) [34], posing no risk of excessive intake. Fe (0.020–31.90 µg/g dw) and Ni (3.64–32.39 µg/g dw) also showed noticeable variation across samples. The low Fe levels may reflect poor aqueous solubility or phytate binding [35], while Ni levels aligned with pumpkin seed oil values [36]. Hull-less varieties SK and SKR had the lowest Ni levels, and estimated intakes remained well below the permitted daily intake [33]. In contrast to earlier findings [35], Mn was undetectable, while Co was consistently absent, in line with previous reports [30]. Phosphorus was below LOQ in all samples, despite its known abundance in pumpkin seeds [30], likely due to insoluble forms such as phytates. These findings underscore the importance of systematic mineral profiling for quality control, especially in therapeutic applications.

Metabolite profiling revealed a diverse phytochemical composition across samples, with consistent detection of Cuc and nucleosides, aligning with the previous literature [37,38]. Chlorogenic and caffeic acids were selectively present, while gallic, protocatechuic acids, and flavonoids such as quercetin, catechin, epicatechin, and myricetin were absent. These results are in contrast with those of Macedo et al., who obtained higher yields from ultrasound (UAE) and microwave-assisted (MAE) methods [39], highlighting the impact of extraction technique on phenolic recovery. However, they also noted the absence of apigenin, kaempferol, quercetin, and quercitrin, while only trace amounts of rutin, orientin, and homoorientin were observed in the current study. *p*-Coumaric acid was present in all samples except GA, supporting findings that many phenolics exist in bound forms requiring alkaline hydrolysis for release [40]. Phenolic glycosides (cucurbitosides A-M, except G) were identified in all extracts, consistent with their proposed antioxidant roles [41,42]. Gibberellins were also consistently detected, indicating a conserved biosynthetic pathway in CP [43]. Cucurbitacins (A, D, E, I) were absent, confirming their localization in non-seed tissues [44], though trace cucurbitacin B was found in the LN sample.

Quantitative analysis of carbohydrates, proteins, and free amino acids revealed distinct patterns across the tested cultivars. Sucrose and stachyose emerged as the dominant carbohydrates, reaching maximum concentrations of 268.63 ± 4.87 mg/g dw and 117.00 ± 0.94 mg/g dw, respectively, in the SKR sample. Verbascose and raffinose were also consistently detected across all samples in lower concentrations, indicating a saccharide profile enriched in raffinose family oligosaccharides (RFOs), typically associated with seed storage [45]. While direct comparisons to whole seed composition are limited by methodological differences, the literature provides context: Mansour et al. reported high levels of sucrose (17.9 mg/g dw), stachyose (8.1 mg/g dw), and raffinose (4.1 mg/g dw) in defatted CP seed meal [46]. Similarly, Polyzos et al. found sucrose as the predominant carbohydrate in *C. maxima* seeds and seed cake (1.97–2.90 g/100 g dw), with much lower levels of trehalose, glucose, and fructose, and undetectable levels of raffinose and stachyose [47]. Glu and Arg were the most abundant amino acids, consistent with findings by Horak et al., who also reported high levels of Arg, Glu, and Ala in CP var. *styriaca* pressed cake [48]. The available literature links both Arg and Glu to cardiovascular modulation, while Cuc has been associated with antiparasitic activity [49]. Arg further contributes to lipid-lowering and vascular effects, particularly when delivered in the natural seed matrix [50]. Glu and Ala, notably high in SKR, support neurotransmission and nitrogen metabolism [32]. The distinct clustering of SK and SKR in PCA and hierarchical analysis reflects major differences in free amino acid and total protein content. Despite SKR having 47% less protein than SK, it showed markedly higher levels of nearly all amino acids, especially Glu, Arg, Ala, and Cuc, and a higher total nitrogen content (34.48%), indicating a shift toward free amino acids. As the quantification of free amino acids used 0.1 N HCl with limited impact on protein hydrolysis [51], this shift likely results from endogenous protein degradation or inherent varietal traits. While storage-induced proteolysis is a possible factor [52], similar saccharide profiles and unchanged total carbohydrate content suggest that pre-harvest conditions or genetic variation are more likely responsible for the differences.

Trigonelline, a hydrophilic pyridine alkaloid found in fenugreek and green coffee beans, is known for its antimicrobial, anticancer, and antihyperglycemic properties, and showed a strong safety profile in preclinical studies [53]. Li et al. reported trigonelline levels of 12.8–23.0 mg/100 g in CP seeds and pulp [54]. In our extracts, trigonelline ranged from 0.41 ± 0.01 to 3.45 ± 0.03 mg/g dw, equating to a safe daily intake of 0.14–3.02 mg based on proposed extract doses (350–875 mg). Based on the similarity in molecular formula of both trigonelline and p-aminobenzoic acid (PABA), we further investigated if PABA is present in our extracts. The absence of a second peak at *m*/*z* 138.055 confirmed that, between the two compounds, only trigonelline is present in the extracts. However, Appendino et al. have identified PABA as part of triterpenoid esters such as zucchini factor A and related multiflorane derivatives in the seeds of *Cucurbita* species [55,56]. These compounds include PABA covalently bound to complex triterpenoid scaffolds that are unlikely to be extracted or detected under our experimental conditions which were optimized for polar metabolites.

Lignan levels varied slightly, with higher concentrations in RZ, SS, and GA. Their absence in hull-less varieties (SK, SKR) may reflect limitations in extraction conditions, such as polarity or lack of hydrolysis, as Sicilia et al. detected Seco and Lar in CP var. *styriaca* using ethanolic extraction followed by hydrolysis [57]. Similarly, Milder et al. reported the presence of Lar (64 µg/100 g), Pino (37 µg/100 g), and Seco (18 µg/100 g) in CP. var *melopepo *seeds, following a hydro-methanolic extraction under mild alkaline conditions [58].

Overall, the comparative analysis revealed clear compositional differences across extract samples. Carbohydrates were the dominant constituents followed by proteins and free amino acids, while trigonelline and lignans were consistently low. Free amino acid levels varied considerably, with SKR and SW showing the highest content, and GA, GV, and SS showing the lowest content.

These findings underscore the necessity for a broader quality control framework that integrates both lipophilic and hydrophilic constituents to ensure a comprehensive assessment of extract composition and consistency across CP cultivars. Our results reveal substantial compositional variability of the extracts influenced by seed morphology and varietal differences, particularly in the content of amino acids, carbohydrates, trigonelline, and lignans. Based on our analyses, trigonelline and lignans may serve as useful markers for distinguishing between cultivars. The extract variability highlights the importance of detailed profiling, especially of lignans and other secondary metabolites, in future studies to better understand their contribution to extract quality and pharmacological potential.

While these findings provide important insights, several methodological limitations should be considered when interpreting the results. First, analyses were conducted on technical rather than biological replicates, which may limit the broader applicability of the findings across different cultivation conditions. Second, complete taxonomic identification of all CP varieties was not possible due to inconsistent labeling provided by seed suppliers, potentially affecting the reproducibility of cultivar specific comparisons. Third, the PCA analysis included only quantitatively determined compounds, excluding metabolites detected at trace levels, which may contribute to chemical variability and possibly to biological activity. Finally, as this study did not aim to identify pharmacologically active constituents, no functional assays were performed, warranting future studies integrating bioactivity-guided fractionation or receptor-based assays to assess the therapeutic relevance of specific hydrophilic components.

Importantly, our findings challenge the notion of treating CP as a uniform botanical entity. The substantial phytochemical diversity observed across varieties indicates that CP is not necessarily CP in a chemical or therapeutic sense. Therefore, monographs and regulatory texts should be revised or interpreted with caution, clearly specifying the CP variety used to avoid ambiguity in research, clinical application, and quality assurance.

## 4. Materials and Methods

### 4.1. Chemicals and Reagents

Common solvents, and general-purpose reagents used throughout the study, are listed in Appendix B. The section below details the reference substances and standards employed for compound identification and quantification. (–)-Secoisolariciresinol (cat. no. 80532), secoisolariciresinol diglucoside (cat. no. 80533), (+)-pinoresinol (cat. no. 89525), pinoresinol-4-O-glucoside (cat. no. 84251), pinoresinol diglucoside (cat. no. 89850), (−)-matairesinol (cat. no. 80497), olivil (cat. no. 83883), cucurbitacin A (cat. no. 84046), cucurbitacin B (cat. no. 82226), cucurbitacin D (cat. no. 80254), cucurbitacin E (cat. no. 80013), cucurbitacin E-2-*O*-glucoside (cat. no. 80613), cucurbitacin I (cat. no. 89464), cucurbitin chloride 58% (cat. no. 80034), vitexin-2-glucoside (cat. no. 84193), orientin (cat. no. 83529), vitexin-2-rhamnoside (cat. no. 89291), protocatechuic acid (cat. no. 89766), and epicatechin (cat. no. 89192) were obtained from Phytolab GmbH & Ko KG (Vestenbergsgreuth, Germany). (+)-Isolariciresinol (cat. no. CFN98915) was from ChemFaces (Wuhan, China), while (+)-lariciresinol (cat. no. 06892), (−) olivil 4″-*O*-glucoside (cat. no. SMB0026), naringin (cat. no. 71162), *p*-coumaric acid (cat. no. C9008), glucose (cat. no. 49139), fructose (cat. no. F0127), sucrose (cat. no. S9378), raffinose (cat. no. R0250), stachyose (cat. no. S4001), verbascose (cat. no. 56217), alanine (cat. no. A7627), tryptophan (cat. no. T0254), asparagine (cat. no. A0884), glutamine (cat. no. 49419), aspartic acid (cat. no. A8949), glutamic acid (cat. no. G1251), arginine (cat. no. A5131), ethanolamine (cat. no. E9508), gamma-aminobutyric acid (cat. no. A2129), nicotinic acid (cat. no. 72309), guanosine (cat. no. G6752), and inosine (cat. no. I4125) were obtained from Sigma-Aldrich (St. Louis, MO, USA). Vitexin (cat. no. 1235S), epicatechin gallate (cat. no. 0978S), homoorientin (cat. no. 1055S), epigallocatechin (cat. no. 0979S), quercitrin (cat. no. 1236S), isoquercitrin (cat. no. 1327S), myricetin (cat. no. 1127S), kaempferol (cat. no. 1124S), apigenin (cat. no. 1102S), myrcitrin (cat. no. 1029S), and 8-hydroxypinoresinol *O*-beta D-glucopyranoside (cat. no. SMB00145) were from Extrasynthese (Genay, France). Amino Acid Standard H (a mixture of 18 amino acids, cat. no. 20088) and Pierce™ BCA Protein Assay Kit (cat. no. A55865 containing BCA Reagent A, 500 mL; BCA Reagent B 25 mL, and prediluted Albumin Standard) were from Thermo Fisher Scientific (Waltham, MA, USA), while AccQ-Tag Ultra Derivatization Kit (cat. no. 186003836, containing AccQ-Tag Ultra Borate Buffer, AccQ-Tag Ultra Reagent Powder in Vial 1 A, and AccQ-Tag Ultra Reagent Diluent in Vial 2B) was from Waters (Milford, MA, USA).

### 4.2. Plant Material

**Figure 9:**
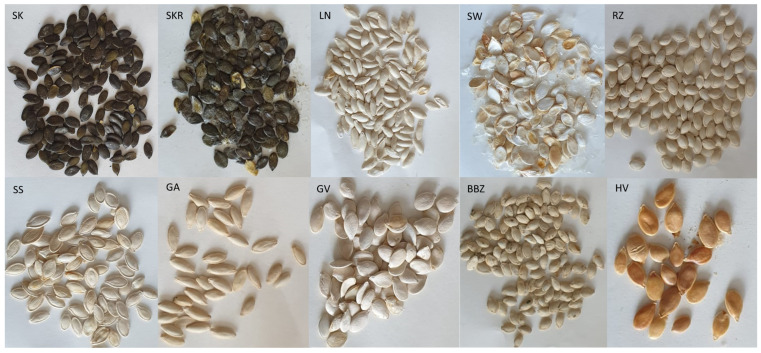
*Cucurbita pepo* (CP) seeds used in the study. Sample codifications are as follows: SK (CP convar. *citrullina* var. *styriaca*), SKR (CP var. *styriaca* cultivar Gleisdorfer Rustikal), LN (CP—Lady Nail), SW (CP—Snow White), RZ (CP var. *giromontia*—Radu), SS (CP—Shine Skin), GA (CP—Greek Cultivar), GV (CP—Grey Volga), BBZ (CP var. *cylindrica*—Black Beauty), HV (CP—Hungarian Cultivar).

The suppliers and characteristics of the dried seeds from ten cultivars of *C. pepo* are detailed in Table 3 and Figure 9. Voucher specimens were deposited in the Department of Pharmacognosy-Phytotherapy, “Grigore T. Popa” University of Medicine and Pharmacy, Iasi, Romania.

### 4.3. Extract Preparation

Hydroethanolic extracts were prepared from each batch of seeds by mixing 3 L of 60% (*v*/*v*) ethanol with 600 g of plant material ground to a particle size of approximately 0.5 mm. The extraction was performed in a 5 L round-bottomed flask placed in a heating mantle (Biobase, Meihua Group Co., Ltd., Jinan, China) equipped with an overhead stirrer (250 rpm) and maintained at 50 °C for 3 h. Following extraction, the solution was filtered, and ethanol was removed under reduced pressure using a rotary evaporator (Büchi R300 Rotavapor, Büchi Labortechnik AG, Flawil, Switzerland). The oil phase obtained after ethanol evaporation was removed by liquid–liquid partitioning in a separatory funnel. The resulting extract was subsequently diluted to 3 L to achieve a final ethanol concentration of 8% (*v*/*v*) and filtered through a 1 μm pore membrane to eliminate residual particulates. The filtrate was further concentrated and freeze-dried (Alpha 1–2 LD plus freeze dryer, Martin Christ Gefriertrocknungsanlagen GmbH, Osterode am Harz, Germany), yielding dry extracts. The freeze-dried extracts were sealed in glass vials, coded according to Table 3, and stored at −20 °C until further use.

### 4.4. Water Content and Elemental Analysis

#### 4.4.1. Water Content

The water content of the samples was determined on a 874 Metrohm oven sample processor (Metrohm, Herisau, Switzerland) coupled to a 917 Coulometer (Metrohm, Herisau, Switzerland), according to the method described by Faria-e-Silva et al. [59]. Results were expressed as wt%.

#### 4.4.2. Metal Elements

Quantitative elemental analysis was performed using FAAS with a contrAA 300 spectrometer (Analytik Jena, Jena, Germany) equipped with a high-resolution Echelle double monochromator and a UV-sensitive charge-coupled device (CCD) detector. A pneumatic concentric jet nebulizer generated the aerosol. Each metal was measured at its optimal analytical wavelength, selected for sensitivity and minimal interference. Calibration curves (0.1–100 mg/L) were constructed from elemental standards. Sample solutions (5 mg/mL in 8% ethanol) were aspirated, aerosolized, and mixed with acetylene/air or acetylene/nitrous oxide, depending on the element.

#### 4.4.3. TC and TN Content

Total organic carbon (TOC), IC, TC, and TN were quantified using a Multi N/C 3100 TOC Analyzer (Analytik Jena, Jena, Germany) with a chemiluminescence detector (CLD) for TN analysis. Samples (25 µg/mL in ultrapure water) were burned in an oxygen rich atmosphere at 800 °C in the presence of a catalyst, converting carbon to CO_2_ and nitrogen containing compounds to NO. Resulting gasses were detected and measured quantitatively. Results were expressed in mg/g of dry extract.

### 4.5. HPTLC Analysis

The analysis of primary and secondary metabolites in the studied samples was initially performed using several HPTLC methods (See Supplementary Materials).

### 4.6. LC-PDA-HRMS Analysis

Phytochemical screening was conducted using LC-PDA-HRMS on a Waters Acquity H-Class UPLC coupled to a SYNAPT G2-S Q-TOF mass spectrometer (Milford, MA, USA) with an ESI source. Analyses were performed in both positive and negative ion modes (100–900 Da), using the following conditions: capillary voltage of 2.5 V, cone voltage of 20 V, source temperature 150 °C, and desolvation temperature 350 °C. Extracts (20 mg/mL in 30% ethanol) were injected (10 µL) and separated on a Waters XSelect HSS T3 column (250 × 4.6 mm, 5 µm) using the following gradient: 0.1% formic acid in water (A), 0.1% formic acid in acetonitrile (B): 0–5 min, 0% B; 5–10 min, 0–5% B; 10–25 min, 5–10% B, 25–50 min, 10–20% B, 50–60 min, 20–40% B, 60–80 min, 40–98% B. The presence of lignans, flavonoids, phenolic acids, nucleosides, epigallocatechin, orientin, vitexin derivatives, quercetin and its glycosides, isolariciresinol, matairesinol, Pino, Lar, Seco, and salicylic acid was evaluated using reference standards (0.1 mg/mL in 70% methanol). Identification was based on retention time, UV spectra, accurate mass, fragmentation, and comparison with standards and the literature using MassLynx 4.1.

### 4.7. Total Carbohydrate Content

The total carbohydrate content was measured using the phenol–sulfuric acid method [60] with a few modifications. Briefly, dry extracts were dissolved in water (1 mg/mL) and a 0.05 mL aliquot was mixed with 0.55 mL water and 1.8 mL sulfuric acid in a heat-resistant tube, followed by the immediate addition of 0.36 mL of 5% (*m*/*v*) phenol solution. After vortexing (5 s), the mixture was incubated at 90 °C for 5 min and cooled. The absorbance was measured at 590 nm using a Specord 210 plus UV-Vis spectrophotometer equipped with ASpect UV 2.0 software (Analytik Jena, Jena, Germany). A calibration curve prepared under the same conditions with D-glucose as standard (0.1–1 mg/mL) was used for quantification.

### 4.8. UPLC Analysis of Saccharides

Monosaccharides and oligosaccharides were analyzed using a Waters Acquity H-Class UPLC system coupled with a 1260 Infinity II ELSD (Agilent, Santa Clara, CA, USA). Separation was performed on a Phenomenex Luna Omega Sugar column (150 × 3.6 mm, 3 µm) following the manufacturer’s guidelines with minor modifications [61]. The mobile phases used were water (A) and acetonitrile–isopropanol–water, 90:5:5, *v*/*v*/*v* (B). Two separate isocratic methods were used, each operating at a flow rate of 1 mL/min. Monosaccharides were separated using an A–B ratio of 8:92 for 18 min, while oligosaccharides were analyzed using an A–B ratio of 29:71 for 17 min. Samples (5 and 10 mg/mL in 50% acetonitrile, respectively) were injected (10 µL) and the column temperature was maintained at 30 °C. ELSD settings included an evaporator temperature of 90 °C, nebulizer temperature of 40 °C, and gas flow rate of 1.5 L/min. Compound identification was achieved by comparing retention times with those of reference standards using Empower 3 software (Waters, Milford, MA, USA). Calibration curves were prepared using standard solutions (0.01–2.00 mg/mL) of rhamnose, glucose, fructose, sucrose, raffinose, stachyose, and verbascose, all dissolved in 50% acetonitrile.

### 4.9. Total Protein Content

A Spectro Star Nano microplate reader equipped with reader control software (BMG LABTECH, Ortenberg, Germany) was employed for the total protein quantification using the BCA assay kit, according to the manufacturer’s protocol [62]. Briefly, 25 µL of extract (2.5 mg/mL in PBS) was mixed with 200 µL of BCA working reagent and incubated at 37 °C for 30 min. After cooling for 5 min at room temperature, the absorbance was measured at 562 nm. Total protein content was expressed as mg bovine serum albumin (BSA) equivalents per gram of extract (mg BSA/g extract).

### 4.10. HPLC Analysis of Free Amino Acids

Free amino acids in CP seed extracts were analyzed using an Acquity H-Class UPLC system with PDA and QDA detectors following derivatization with the ACCQ-Tag Ultra kit (Waters, Milford, MA, USA), per the manufacturer’s protocol [63]. Extracts (10 mg/mL in 0.1 N HCl) were derivatized with 70 µL borate buffer and 20 µL reagent, vortexed, incubated at room temperature, then heated at 55 °C for 10 min. After centrifugation (5000 rpm, 10 min), 2 µL of the supernatant was injected. The derivatizing agent, 6-aminoquinolyl-N-hydroxysuccinimidyl carbamate (ACQ), binds to primary and secondary amines to form stable carbamate derivatives with peak absorbance at 248 nm. Excess reagent reacts with water to form 6-aminoquinoline (AMQ), which can form bis-derivatives.

Analytes were identified by their retention times and corresponding *m*/*z* values using reference standards. All compounds produced single peaks, except Cuc, which formed both single- and double-derivatized products due to its two reactive amino groups. Separation was performed on a Waters BEH C18 column (150 × 2.1 mm, 1.7 µm) at 45 °C, with a flow rate of 0.4 mL/min. A gradient elution was applied using mobile phase A (ammonium acetate, acetonitrile, acetic acid, water; pH 4.3) and B (acetonitrile) following the program 0–1.35 min, 0.5–0.5% B; 1.35–18 min, 0.5–8% B; 18–21 min, 8–19.5% B; 21–24 min, 19.5% B, 24–24.5 min, 19.5–59% B; 24.5–26 min, 59% B, 26–26.5 min, 59–0.5% B, 26.5–33 min, 0.5% B. Calibration curves were built using a standard mix of 18 amino acids (2.5 µmol/mL), and for additional compounds (Asp, Glu, Trp, Cuc, GABA, Eta), individual standards were prepared (0.6–0.005 mg/mL in 0.1 N HCl).

### 4.11. UPLC Analysis of Lignans

Lignans Seco, Lar, Pino were identified and quantified using the same Waters Acquity H-Class UPLC system coupled with PDA and QDA detectors and equipped with a Waters Cortecs C18 column (150 × 3.0 mm, 1.6 µm). Identification was confirmed by comparing the retention time, UV (280 nm) and MS spectra with reference substances, while quantification relied on six-point calibration curves (1–500 µg/mL). The mobile phase consisted of 0.1% formic acid in water (A) and 0.1% formic acid in acetonitrile (B): 0–17.9 min, 15–45% B; 17.9–19.1 min, 45–99% B; 19.1–19.4 min, 99–15% B; 19.4–24 min, 15% B. The gradient ran at a flow rate of 0.4 mL/min, with the column temperature set at 35 °C. Dry extracts (20 mg/mL in 80% methanol) were ultrasonicated for 15 min, and centrifuged at 10,000 rpm for 10 min. Finally, a volume of 2 µL of supernatant was injected.

### 4.12. UPLC Analysis of Trigonelline

Trigonelline identification and quantification was conducted using the same system and detectors as mentioned in Section 4.10. Chromatographic separation was performed on a Waters X Select HSS T3 column (250 × 4.6 mm, 5 µm) maintained at room temperature (24.5 °C). The mobile phase consisted of water with 0.1% formic acid (A) and acetonitrile with 0.1% formic acid (B). The gradient program started with an isocratic step for 7 min at 0% B; 7–10 min, 0–98% B; 10–20 min, 98% B; 20–22 min, 98–0% B; 22–32 min, 0% B. Samples (25 mg/mL in 8% ethanol) were injected (5 µL) and separated at a flow rate of 0.8 mL/min. Quantification was carried out using a six-point calibration curve (1–500 µg/mL). The UV detection was set at 263 nm, whereas a single ion recording (SIR) channel was set at *m/z* 138. A manual splitter directed 25% of the flow to the QDa detector.

### 4.13. Multivariate Analysis

Quantitative phytochemical analyses were performed for each sample in triplicate and results were reported as mean ± standard deviation (SD). A multivariate analysis was used to investigate the quantitative composition of the hydrophilic extracts of CP seeds. PCA was performed to reduce dimensionality and identify patterns in the chemical composition of the tested extracts. The PCA considered 35 quantified variables, including total protein, individual sugars, free amino acids, and secondary metabolites. The dataset was standardized using a Z-score standardization, and the principal components were computed, together with their eigenvalues, proportion of variance, and cumulative variance, to determine the optimal number of components for an accurate interpretation. This analysis was carried out using GraphPad Prism software version 10.04 (GraphPad Software, Boston, MA, USA) and SPSS Statistics version 26.0 (SPSS Inc., Chicago, IL, USA).

## 5. Conclusions

In conclusion, our findings reveal notable variation in the chemical composition of hydroethanolic seed extracts across various CP cultivars, underscoring the need for a comprehensive standardization strategy. While CP convar. *citrullina* var. *styriaca* is currently recommended by the HMPC for use in herbal medicinal products, the Ph. Eur. refers only to hull-less seeded varieties without specifying a cultivar. Our data show that CP var. *styriaca*, cultivar ‘Gleisdorfer Rustikal’ (SKR), exhibits a hydrophilic metabolite profile comparable to that of the reference convar. *citrullina* var. *styriaca* (SK), with even higher levels of certain amino acids and oligosaccharides.

These results highlight the influence of genetic background on extract composition and emphasize the urgent need for regulatory alignment between HMPC and Ph. Eur., including the establishment of a clear and cultivar-specific framework for the standardization of CP seed extracts.

## Figures and Tables

**Table 1:** Levels (mg/g dw) of total organic carbon (TOC), inorganic carbon (IC), total carbon (TC), and total nitrogen (TN) in *Cucurbita pepo* (CP) seed hydrophilic extracts, expressed as mean ± SD (*n* = 3 technical replicates).

Nr.	Sample	TOC [mg/g]	IC [mg/g]	TC [mg/g]	TN [mg/g]
1	SK	449.37 ± 1.05	2.95 ± 0.17	452.32 ± 1.04	14.32 ± 0.02
2	SKR	340.61 ± 3.34	3.68 ± 0.07	344.29 ± 3.34	19.25 ± 0.16
3	LN	394.42 ± 0.68	2.35 ± 0.03	396.77 ± 0.67	16.22 ± 0.07
4	SW	337.48 ± 1.68	2.46 ± 0.01	339.94 ± 1.68	26.57 ± 0.03
5	RZ	397.61 ± 3.08	2.08 ± 0.01	399.69 ± 3.08	14.39 ± 0.18
6	SS	341.83 ± 1.91	4.55 ± 0.03	346.37 ± 1.91	13.32 ± 0.07
7	GA	306.43 ± 0.43	2.31 ± 0.15	308.74 ± 0.40	12.59 ± 0.09
8	GV	528.16 ± 0.75	3.11 ± 0.13	531.27 ± 0.74	13.59 ± 0.02
9	BBZ	297.33 ± 1.95	7.05 ± 0.13	304.38 ± 1.95	14.81 ± 0.14
10	HV	444.47 ± 1.10	3.29 ± 0.24	447.76 ± 1.07	27.09 ± 0.24

Sample codifications are as follows: SK (CP convar. *citrullina* var. *styriaca*), SKR (CP var. *styriaca* cultivar Gleisdorfer Rustikal), LN (CP—Lady Nail), SW (CP—Snow White), RZ (CP var. *giromontia*—Radu), SS (CP—Shine Skin), GA (CP—Greek Cultivar), GV (CP—Grey Volga), BBZ (CP var. *cylindrica*—Black Beauty), HV (CP—Hungarian Cultivar).

**Table 2:** Trigonelline contents expressed as mean (mg/g dw) ± SD (*n* = 3 technical replicates) in the studied hydrophilic extracts of *Cucurbita pepo *(CP) seeds.

Number	Extract	Trigonelline Content [mg/g]	±SD
1	SK	1.764	0.004
2	SKR	1.685	0.002
3	LN	1.765	0.023
4	SW	3.447	0.027
5	RZ	0.459	0.008
6	SS	1.975	0.001
7	GA	0.985	0.002
8	GV	0.413	0.016
9	BBZ	0.407	0.006
10	HV	1.333	0.002

Sample codifications are as follows: SK (CP convar. *citrullina* var. *styriaca*), SKR (CP var. *styriaca* cultivar Gleisdorfer Rustikal), LN (CP—Lady Nail), SW (CP—Snow White), RZ (CP var. *giromontia*—Radu), SS (CP—Shine Skin), GA (CP—Greek Cultivar), GV (CP—Grey Volga), BBZ (CP var. *cylindrica*—Black Beauty), HV (CP—Hungarian Cultivar).

**Table 3:** Description of *Cucurbita pepo* seeds used in the study.

Nr.	Name	Code	Extraction Yield [g]	Country of Origin	Distributor	Batch	Macroscopy
1	*C. pepo* convar. *citrullina*, var *styriaca*	SK	10.25	Austria	Estyria Naturprodukte GmbH	21-039676 0001	Medium, dark green seeds, with a very thin shell and a pointed tip. Length 1.3–1.7 cm, width 0.4–0.6 cm.
2	*C. pepo* var. *styriaca* cultivar Gleisdorfer Rustikal	SKR	10.01	Switzerland	Vitaplant AG	CUP 22_2202	Medium, dark green seeds, with a very thin shell and a pointed tip. Length 1.2–2.1 cm, width 0.5–0.7 cm.
3	*C. pepo*—Lady Nail	LN	11.21	Switzerland	Vitaplant AG	CUP 22_2021	Medium, sharp light-yellow seeds. Length 1.5–2.0 cm, width 0.4–0.5 cm.
4	*C. pepo*—Snow White	SW	15.63	Switzerland	Vitaplant AG	CUP 22_2103	Large, with a thick shell, and light-yellow seeds. Length 2.0–2.5 cm, width 1.0–1.4 cm.
5	*C. pepo* var. *giromontia* -Radu	RZ	10.35	Romania	Agrosem Impex	2MS1196	Small, round, light yellow seeds. Length 0.8–1.5 cm, width 0.5–0.6 cm.
6	*C. pepo*—Shine Skin	SS	13.37	Ukraine	M.A.R. Pumpkin Seeds	CUS18006/20	Medium, with a thick shell, pointed tip, and light-yellow seeds. Length 1.5–2.5 cm, width 0.8–1.0 cm.
7	*C. pepo* -Greek Cultivar	GA	10.21	Greece	Antonio Foods	CUS19001(S/K/H)	Medium, narrow and sharp, light-yellow seeds. Length 1.4–2.1 cm, width 0.4–0.5 cm.
8	*C. pepo*—Grey Volga	GV	12.44	Ukraine	M.A.R. Pumpkin Seeds	CUS18005/20	Medium, with a soft smooth shell, and light-yellow seeds. Length 1.5–2.2 cm, width 0.9–1.0 cm.
9	*C. pepo* var. *cylindrica*—Black Beauty	BBZ	10.33	Romania	Agrosem Impex	2MS1197	Small, round, light yellow seeds. Length 0.9–1.2 cm, width 0.5–0.6 cm.
10	*C. pepo*—Hungarian Cultivar	HV	14.67	Hungary	H.F.I. Hungarian Food Ingredients	CUS18008/20	Medium, round, with a thick orange shell. Length 1.5–2.2 cm, width 0.7–1.0 cm.

## Data Availability

Data are contained within the article and Supplementary Materials.

## Supplementary Materials

The following supporting information can be downloaded at: https://www.mdpi.com/article/10.3390/plants14152308/s1, Figure S1: HPTLC chromatogram of free amino acids; Figure S2: HPTLC chromatogram of saccharides; Figure S3: HPTLC chromatogram of flavonoids and phenolic acids, Table S1: Water content of hydrophilic *Cucurbita pepo* seed extracts determined by Karl Fischer titration (wt%).

## Appendix A

**Table A1:** Qualitative composition of *Cucurbita pepo* hydrophilic extracts using UHPLC-PDA-HRMS.

No.	RT (min)	Compound Name	Chemical Formula	Chemical Class	Calculated Mass	Main Fragment	Ion Type	Other Fragments	Identified	Not Identified	Reference
1	2.47	Cucurbitin	C_5_H_10_N_2_O_2_	amino acid	130.0742	131.0819	[M+H]^+^	85.0766	1–10		[37]
2	4.17	Trigonelline	C_7_H_7_NO_2_	alkaloid	137.0477	138.0557	[M+H]^+^	94.0601	1–10		[54]
3	8.594	Nicotinic acid	C_6_H_5_NO_2_	pyridine-3-carboxylic acid	123.0320	124.0399	[M+H]^+^	106.0271	1–10		[54]
4	14.93	Adenosine	C_10_H_13_N_5_O_4_	nucleoside	267.0968	268.1045	[M+H]^+^	136.0624	1–10		[38]
5	15.37	Guanosine	C_10_H_13_N_5_O_5_	nucleoside	283.0917	282.0841	[M-H]^−^	152.0531 135.0305	1–10		[38]
6	16.32	Gallic acid	C_7_H_6_O_5_	phenolic acids	170.0200	169.0133	[M-H]^−^	125.0236		1–10	[39,64]
7	24.39	Protocatechuic acid	C_7_H_6_O_4_	phenolic acids	154.0266	153.0188	[M-H]^−^	109.0292		1–10	[39,40]
8	25.16	Cucurbitoside L	C_24_H_29_NO_12_	phenolic glycoside	523.1689	522.1614	[M-H]^−^	457.1924 411.1865 122.0247	1–10		[42]
9	34.07	Epigallocatechin	C_15_H_14_O_7_	flavonoid	306.0739	307.0818	[M+H]^+^	289.0715 165.0218		1–10	
10	35.61	Chlorogenic acid	C_16_H_18_O_9_	phenolic acid	354.0951	353.0811	[M-H]^−^	191.0560, 375.0696, 443.0560, 511.0439,	1–3, 5–7, 9	4, 8, 10	[39]
11	35.87	Catechin	C_15_H_14_O_6_	flavonoid	290.0790	289.0715	[M-H]^−^	357.0585, 245.0805		1–10	[39,64]
12	39.41	Cucurbitoside H	C_25_H_31_NO_12_	phenolic glycoside	537.1846	536.1776	[M-H]^−^	383.2079 122.0247	1–10		[41]
13	39.42	Caffeic acid	C_9_H_8_O_4_	phenolic acids	180.0423	179.0349	[M-H]^−^	153.0190, 135.0448, 109.0290	2, 3, 5, 6, 10	1, 2, 4, 7–9	[39,40]
14	40.69	Cucurbitoside M	C_25_H_31_NO_12_	phenolic glycoside	537.1846	536.1776	[M-H]^−^	399.1300 136.0402	1–10		[42]
15	40.98	Gibberelin A 39	C_20_H_26_O_8_	terpenoid	394.1627	393.1555	[M-H]^−^	319.1184, 290.1255, 231.1384	1–10		[43]
16	42.81	Epicatechin	C_15_H_14_O_6_	flavonoid	290.0790	289.0715	[M-H]^−^	245.0808		1–10	[39]
17	44.37	Gibberelin A 8	C_19_H_24_O_7_	terpenoid	364.152	363.1449	[M-H]^−^	275.1650, 257.1543	1–10		[43]
18	44.98	Cucurbitoside D	C_25_H_30_O_13_	phenolic glycoside	538.1686	537.1608	[M-H]^−^	186.1130 137.0236	1–10		[41]
19	46.21	Cucurbitoside I	C_26_H_33_NO_12_	phenolic glycoside	551.2003	550.1920	[M-H]^−^	413.1453	1–10		[42]
20	46.92	Gibberelin A 1	C_19_H_24_O_6_	terpenoid	348.1572	347.1491	[M-H]^−^	241.0126	1–10		[43]
21	48.97	Homoorientin	C_21_H_20_O_11_	flavonoid	448.1006	449.1081	[M+H]^+^	471.0893; 431.0974; 329.0657	4, 5, 7, 9	1–3, 6, 8, 10	
22	49.38	p-coumaric acid	C_9_H_8_O_3_	phenolic acid	164.0473	163.0395	[M-H]^−^	119.0499	1–6, 8–10	7	[39]
23	49.77	Secoisolariciresinol-diglucoside	C_20_H_22_O_6_	lignan	686.2786	327.159	[M+H]^+^	704.3115 295.1328 163.0755 137.0594	3–7, 9, 10	1, 2, 8	
24	50.42	Orientin	C_21_H_20_O_11_	flavonoid	448.1006	449.1075	[M+H]^+^	431.0966 329.0656	4, 5, 7, 9	1–3, 6, 8, 10	
25	48.70	Hydroxypinoresinol glucopyranoside	C_26_H_32_O_12_	lignan	536.1894	559.1791 (Na adduct)	[M+H]^+^	163.0754	3, 10	1, 2, 4–9	
26	51.09	Cucurbitoside B	C_26_H_32_O_13_	phenolic glycoside	552.1843	551.1759	[M-H]^−^	137.0234	1–10		[41]
27	51.76	Vitexin-2-glucoside	C_27_H_30_O_15_	flavonoid glycoside	594.1585	595.1657	[M+H]^+^	617.1475, 475.3251 453.3431		1–10	
28	53.84	Rutin	C_27_H_30_O_16_	flavonoid	610.1534	609.1453	[M-H]^−^	677.1335	1, 5–8	2–4, 9, 10	[39,64]
29	54.71	Olivil	C_20_H_24_O_7_	lignan	376.1522	399.1412 (Na adduct)	[M+H]^+^	341.1387 323.1279 187.0756	3–5, 7–9	1, 2, 6, 10	
30	55.33	Vitexin-2-rhamnoside	C_27_H_30_O_14_	flavonoid glycoside	578.1636	579.171	[M+H]^+^	601.1523		1–10	
31	55.36	Vitexin	C_21_H_20_O_10_	flavonoid	432.1056	433.1129	[M+H]^+^	415.1014		1–10	[39]
32	54.41	Myrcitrin	C_21_H_20_O_12_	flavonoid	464.0955	463.087	[M-H]^−^	509.0938	1, 6	2–5, 7–10	
33	54.84	Cucurbitoside K	C_23_H_34_O_12_	phenolic glycoside	502.205	501.1969	[M-H]^−^	399.1287 123.0447	1–10		[42]
34	54.91	Hyperoside	C_21_H_20_O_12_	flavonoid	464.0955	463.0876	[M-H]^−^	301.0354, 151.0014		1–10	
35	55.12	Epicatechin gallate	C_22_H_18_O_10_	flavonoid	442.09	441.0819	[M-H]^−^	473.0710		1–10	
36	55.67	Isoquercitrin	C_21_H_20_O_12_	flavonoid	464.0955	463.0871	[M-H]^−^	301.0324, 151.0018		1–10	
37	56.38	Cucurbitoside C	C_25_H_30_O_12_	phenolic glycoside	522.1737	521.1652	[M-H]^−^	121.0291	1–10		[41]
38	54.47	Cucurbitoside E	C_24_H_28_O_12_	phenolic glycoside	508.1581	507.1497	[M-H]^−^	121.0291	1–10		[41]
39	57.50	Naringin	C_27_H_32_O_14_	flavanone 7-O glycoside	580.1792	579.1414	[M-H]^−^	647.1518		1–10	[39,64]
40	57.58	Cucurbitoside J	C_25_H_38_O_13_	phenolic glycoside	546.2312	545.2236	[M-H]^−^	353.1692	1–10		[42]
41	57.70	Cucurbitoside A	C_26_H_32_O_12_	phenolic glycoside	536.1894	535.1805	[M-H]^−^	413.1449 347.1494 121.0295	1–10		[41]
42	57.72	Quercitrin	C_21_H_20_O_11_	flavonoid	448.1006	447.0929	[M-H]^−^	303.0469		1–10	
43	58.18	Cucurbitoside F	C_23_H_36_O_12_	phenolic glycoside	516.2207	515.2123	[M-H]^−^	413.1451 137.0606	1–10		[42]
44	59.78	Myricetin	C_15_H_10_O_8_	flavonoid	318.0297	317.0298	[M-H]^−^	287.0165		1–10	[64]
45	59.87	Secoisolariciresinol	C_20_H_26_O_6_	lignan	362.1729	327.1593	[M+H]^+^	385.1620 295.1328 163.0758 137.0598	3–10	1, 2	[65]
46	60.15	Salicylic acid	C_7_H_6_O_3_	carboxylic acid	138.0317	137.0239	[M-H]^−^	297.0372	1–10		[66]
47	60.80	Lariciresinol	C_20_H_24_O_6_	lignan	360.1573	219.1019	[M+H]^+^	189.0912 721.1034	3–10	1,2	[57]
48	63.37	Daidzein	C_15_H_10_O_4_	isoflavone	254.0579	253.0504	[M-H]^−^	223.0413		1–10	[65]
49	59.29	Quercetin	C_15_H_10_O_7_	flavonoid	302.0427	301.0349	[M-H]^−^	179.0108 125.0114		1–10	[64]
50	63.94	Isolariciresinol	C_32_H_46_O_16_	lignan	360.1573	361.1646	[M+H]^+^	209.0814		1–10	
51	64.96	Pinoresinol	C_20_H_22_O_6_	lignan	358.1416	341.1385	[M+H]^+^	323.1273 175,0756	3–10	1, 2	[67]
52	65.16	Cucurbitacin E-2-O glucoside	C_38_H_54_O_13_	cucurbitacin type triterpene	718.3564	741.3465	[M+H]^+^	479.2796 461.2689	3	1, 2, 4–10	[67]
53	65.86	Cucurbitacin A	C_32_H_46_O_9_	cucurbitacin type triterpene	574.3142	597.3035	[M+H]^+^	515.3007 467.2791		1–10	[67]
54	65.60	Apigenin	C_15_H_10_O_5_	flavonoid	270.0528	271.0609	[M+H]^+^	158.0123		1–10	[39]
55	66.49	Cucurbitacin D	C_30_H_44_O_7_	cucurbitacin type triterpene	516.3087	539.2978	[M+H]^+^	481.2951 499.3055		1–10	[44]
56	66.50	Kaempferol	C_15_H_10_O_6_	flavonoid	286.0477	285.0402	[M-H]^−^	164.9982 119.0323		1–10	[64]
57	68.14	Cucurbitacin I	C_30_H_42_O_7_	cucurbitacin type triterpene	514.2931	537.2821	[M+H]^+^	97.2896 479.2794 461.2686		1–10	[44]
58	71.11	Cucurbitacin B	C_32_H_46_O_8_	cucurbitacin type triterpene	558.3193	581.3089	[M+H]^+^	481.295 463.2846	3	1, 2, 4–10	[44]
59	75.51	Matairesinol	C_20_H_22_O_6_	lignan	358.1416	359.2412	[M+H]^+^	375.2146 175.1334 103.0756	1–10		

## Appendix B. List of Routine Solvents and General Laboratory Reagents

Acetonitrile (LC-MS grade cat. no. AE70.2) was provided by Roth (Karlsruhe, Germany); acetonitrile HPLC grade (cat. no. 83639.329), ethanol absolute (cat. no.20821.330), methanol (cat. no. 20864.420), LC-MS grade formic acid (cat. no. 84865.260), ethyl acetate (cat. no. 83621.320), and isopropanol (cat. no. 20842.330) were purchased from VWR (Radnor, PA, USA). Toluene (cat. no. 1.08325.2500), 1-butanol (cat. no. 1.01990.2500), boric acid (cat. no. 1.00165.0100), ninhydrin (cat. no. 1.06762.0010), disodium hydrogen phosphate hydrate (cat. no. 1.06580.0500), phenol (cat. no. 16016), and sulfuric acid (cat. no. 1007131) were from Merck (Darmstadt, Germany). Ultrapure water was obtained from ELGA Ultrapure water system PURELAB^®^ 7120 (ELGA LabWater, High Wycombe, UK).
