# Supplementary material for: Profiling Hydrophilic Cucurbita pepo Seed Extracts: A Study of European Cultivar Variability
Source: Plants (Basel). 2025 Jul 26;14(15):2308. doi: 10.3390/plants14152308 (PMC12348540; doi:10.3390/plants14152308)
Supplement: Supplementary file 1 [file plants-14-02308-s001.zip › plants-3707923-supplementary.pdf]

Article

Profiling Hydrophilic *Cucurbita pepo* Seed Extracts: A Study of European Cultivar Variability

Adina-Elena Grasu[1], Roman Senn[2], Christiane Halbsguth[2], Alexander Schenk[2], Veronika Butterweck[2,*] and Anca Miron[1,*]

[1] Department of Pharmacognosy-Phytotherapy, Faculty of Pharmacy, Grigore T. Popa University of Medicine and Pharmacy, Universitatii Str. 16, 700115 Iasi, Romania; adina-elena.grasu@d.umfiasi.ro (A.-E.G.)
[2] Medical Department, Max Zeller & Söhne AG, Seeblickstrasse 4, 8590 Romanshorn, Switzerland; roman.senn@zellerag.ch (R.S.); christiane.halbsguth@zellerag.ch (C.H.); aschenk58@icloud.com (A.S.)
* Correspondence: veronika.butterweck@zellerag.ch (V.B.), anca.miron@umfiasi.ro (A.M.)

4.5. HPTLC analysis

Free amino acids

To investigate the free amino acid profile, the dry extracts were dissolved in 50 mM sodium acetate solution (10 mg/mL), ultrasonicated for 15 min, and centrifuged at 10,000 rpm for 10 min. Each sample (10 µL) was applied as 8 mm band using the Automatic TLC sampler 4 (CAMAG, Muttenz, Switzerland), on a HPTLC silica gel 60 F_{254} plate (10 cm × 20 cm, Merck, Darmstadt, Germany). Reference standards (0.5 mg/mL in water) were applied as 5 µL bands. The plate was placed in the Automatic Development Chamber 2 (CAMAG, Muttenz, Switzerland), dried for 5 min, pre-conditioned for 10 min at 33% relative humidity with $MgCl_2$, and developed to a distance of 85 mm in a chamber pre-saturated with the mobile phase for 20 min. The mobile phase consisted of ethyl acetate:methanol:citric acid–phosphate buffer 3:3:4 (v/v/v). After development, the plate was dried for 5 min in the Automatic Development Chamber 2, derivatized by dipping in ninhydrin reagent (1.5 g of ninhydrin dissolved in a mix of 500 mL of *n*-butanol and 15 mL of acetic acid) for 2 s, and heated at 120 °C for 5 min using a TLC plate heater III (CAMAG, Muttenz, Switzerland).

Carbohydrates

The HPTLC fingerprint of carbohydrates was performed on the same equipment. Extracts were dissolved in 50% aqueous acetonitrile (v/v) to a concentration of 10 mg/mL, sonicated, and centrifuged as described above. Supernatants (10 µL) were applied as 8 mm bands on a HPTLC silica gel 60 F_{254} plate (10 cm × 20 cm, Merck, Darmstadt, Germany). A mixture of reference carbohydrates including sucrose, raffinose, stachyose, and verbascose, was prepared (0.5 mg/mL of each in 50% acetonitrile); 1 µL of this mixture was applied on the HPTLC plate. The latter was further activated at 33% relative humidity and developed to a distance of 85 mm using a mobile phase composed of ethyl acetate:methanol:boric acid (5 mg/mL):acetic acid 50:40:10:2 (v/v/v/v) in an unsaturated chamber. After drying, the plate was derivatized by

immersion in a diphenylamine-aniline derivatization reagent, prepared by dissolving 2 g of diphenylamine and 1 mL of aniline in 80 mL of methanol, followed by the addition of 10 mL of orthophosphoric acid (85%). The mixture was shaken until clear and diluted with 10 mL of methanol.

Phenolic acids and flavonoids

To investigate the presence of flavonoids and phenolic acids, 10 µL of each sample (10 mg/mL in 80% methanol, v/v) were applied as 8 mm bands onto the same type of plate. A mixture of flavonoids and phenolic acids including rutin, hyperoside, chlorogenic acid, and rosmarinic acid was prepared (0.25 mg/mL of each in 80% methanol) and 1 µL of the mixture was applied as bands. The plate was developed with ethyl acetate:water:formic acid:acetic acid 100:26:11:11 (v/v/v/v), heated for 10 min at 100 °C, sequentially immersed in 1% diphenylboric acid-2-aminoethylester in methanol and 5% polyethylene glycol 400 in methanol, and examined under UV light at 365 nm.

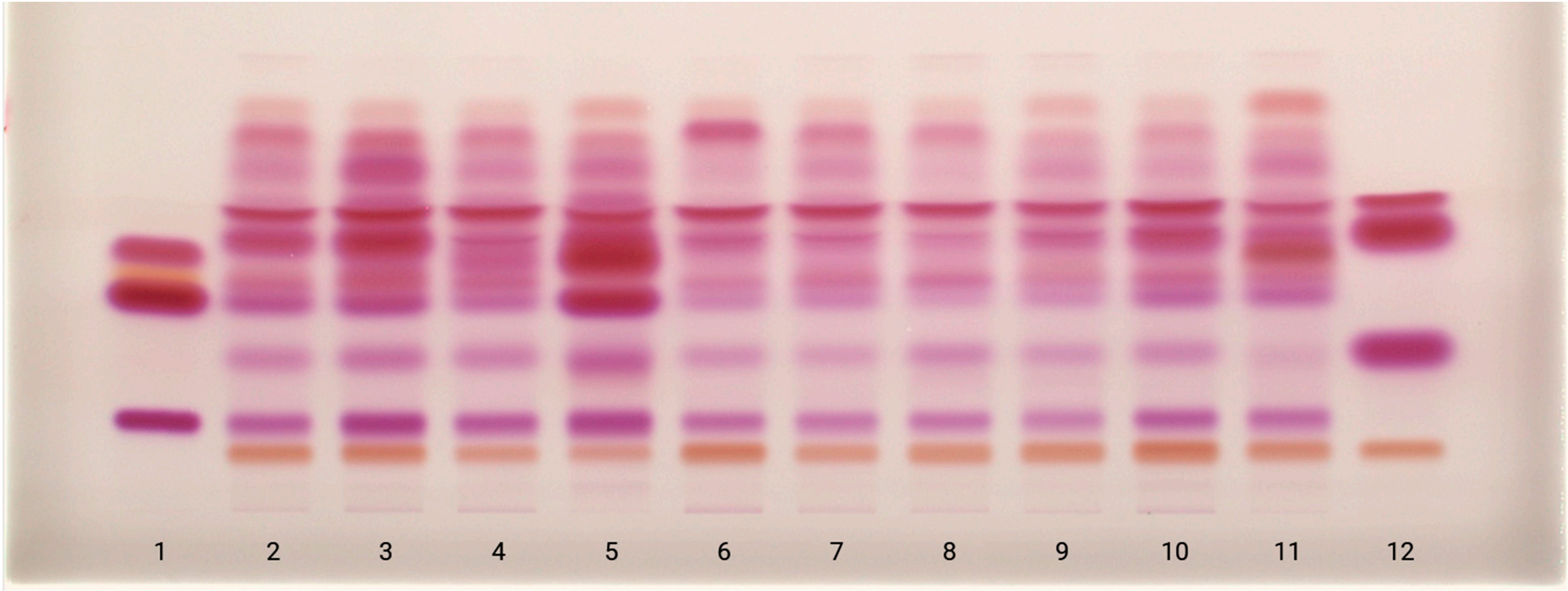

Figure S1. HPTLC chromatogram of free amino acids present in *Cucurbita pepo* (CP) seed hydrophilic extracts (10 mg/mL in 50 mM sodium acetate solution): **1.** mix of: L-arginine, gamma-aminobutyric acid, L-asparagine, and L-glutamine (from bottom to top); **2.** SK (CP convar. *citrullina* var. *styriaca*); **3.** SKR (CP var. *styriaca* cultivar Gleisdorfer Rustikal); **4.** LN (CP - Lady Nail); **5.** SW (CP - Snow White); **6.** RZ (CP var. *giromontia* - Radu); **7.** SS (CP - Shine Skin); **8.** GA (CP - Greek Cultivar); **9.** GV (CP - Grey Volga); **10.** BBZ (CP var. *cylindrica* - Black Beauty); **11.** HV (CP - Hungarian Cultivar); **12.** mix of: cucurbitin, ethanolamine, L-alanine, and glutamic acid (from bottom to top).

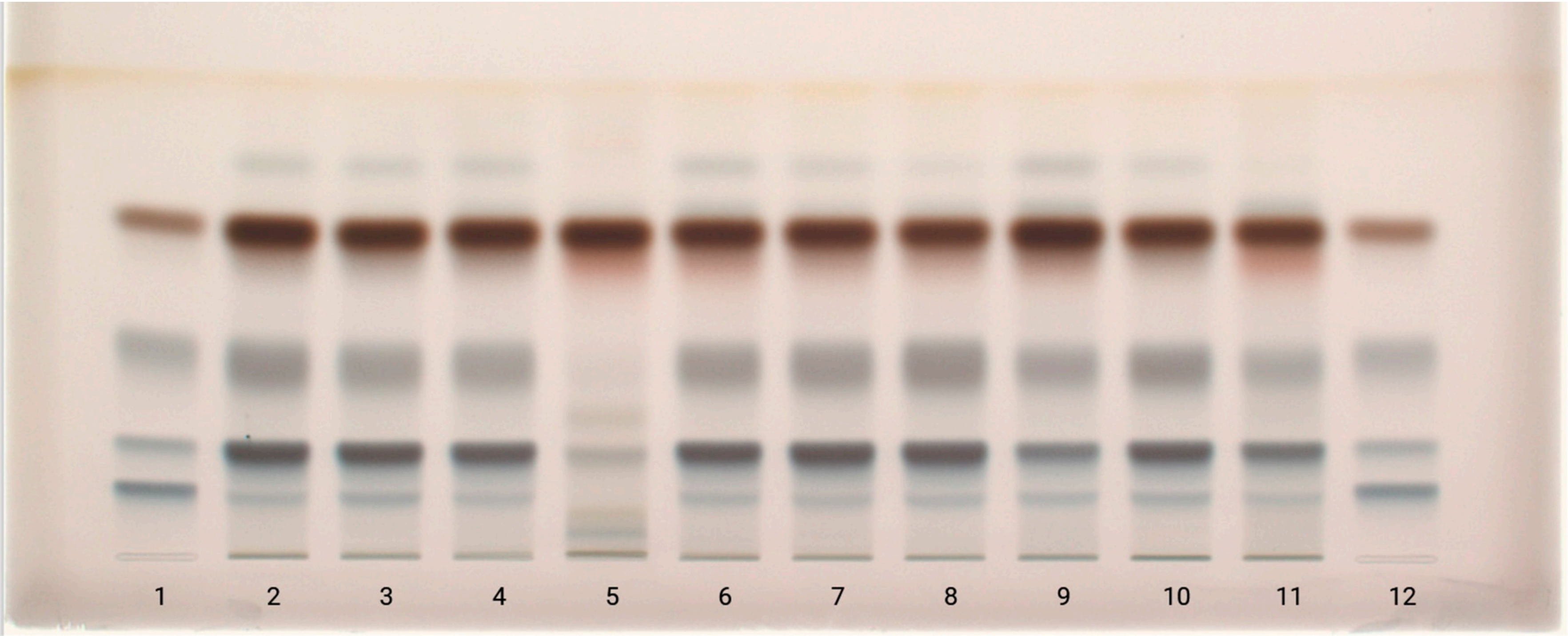

Figure S2. HPTLC chromatogram of carbohydrates present in *Cucurbita pepo* (CP) seed hydrophilic extracts (10 mg/mL in 50 % acetonitrile): **1.** mix of: verbascose, stachyose, raffinose, and sucrose (from bottom to top); **2.** SK (CP convar. *citrullina* var. *styriaca*); **3.** SKR (CP var. *styriaca* cultivar Gleisdorfer Rustikal); **4.** LN (CP - Lady Nail); **5.** SW (CP - Snow White); **6.** RZ (CP var. *giromontia* - Radu); **7.** SS (CP - Shine Skin); **8.** GA (CP - Greek Cultivar); **9.** GV (CP - Grey Volga); **10.** BBZ (CP var. *cylindrica* - Black Beauty); **11.** HV (CP - Hungarian Cultivar); **12.** mix of: verbascose, stachyose, raffinose, and sucrose (from bottom to top).

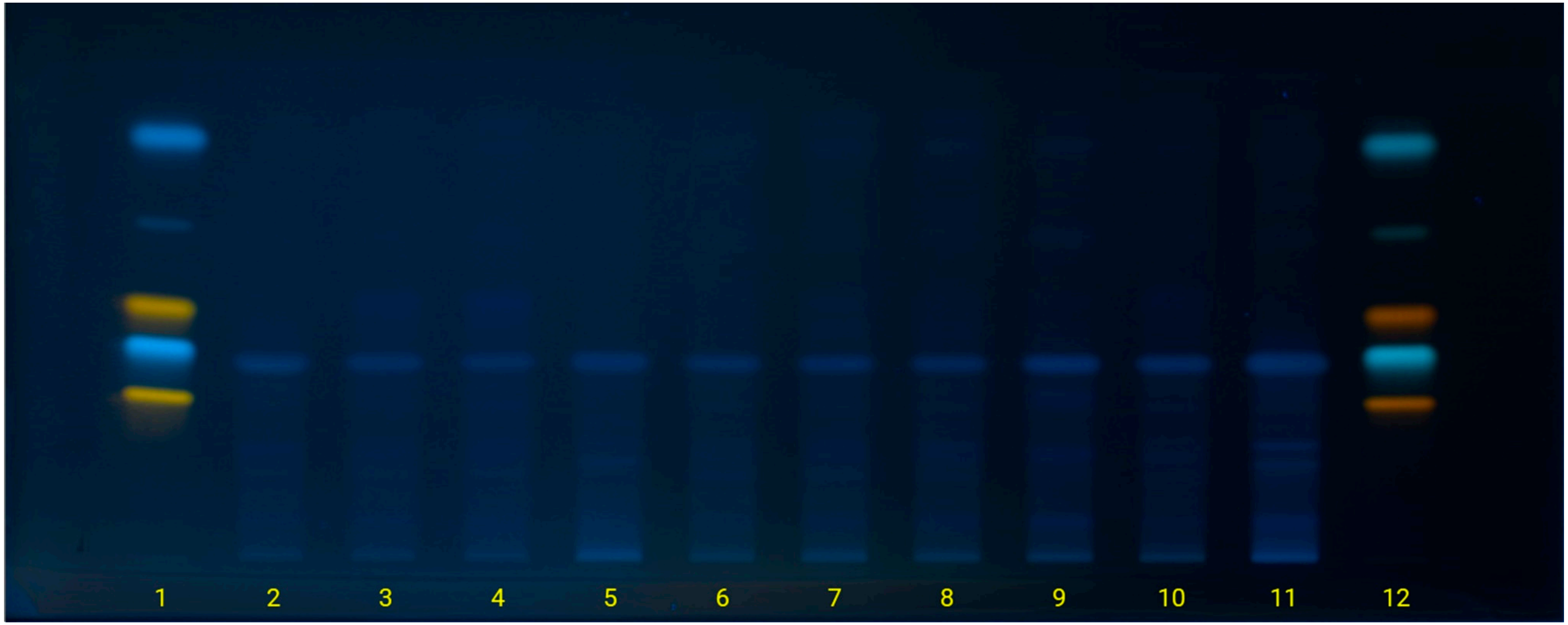

Figure S3. HPTLC chromatogram of flavonoids and phenolic acids present in *Cucurbita pepo* (CP) seed hydrophilic extracts (10 mg/mL in 80 % methanol) **1**. mix of rutin, chlorogenic acid, hyperoside, and rosmarinic acid (from bottom to top); **2.** SK (CP convar. *citrullina* var. *styriaca*); **3.** SKR (CP var. *styriaca* cultivar Gleisdorfer Rustikal); **4.** LN (CP - Lady Nail); **5.** SW (CP - Snow White); **6.** RZ (CP var. *giromontia* - Radu); **7.** SS (CP - Shine Skin); **8.** GA (CP - Greek Cultivar); **9.** GV (CP - Grey Volga); **10.** BBZ (CP var. *cylindrica* - Black Beauty); **11.** HV (CP - Hungarian Cultivar); **12**. mix of rutin, chlorogenic acid, hyperoside, and rosmarinic acid (from bottom to top).

Table S1. Water content of hydrophilic *Cucurbita pepo* (CP) seed extracts determined by Karl Fischer titration (wt%)

Number	Extract	Water content [wt%]	± SD
1.	SK	2.393	0.176
2.	SKR	4.956	0.265
3.	LN	4.903	0.054
4.	SW	11.847	0.594
5.	RZ	4.846	0.213
6.	SS	4.887	0.296
7.	GA	3.837	0.007
8.	GV	3.343	0.158
9.	BBZ	3.697	0.121
10.	HV	4.610	0.369

Sample codifications are as follows: SK (CP convar. *citrullina* var. *styriaca*), SKR (CP var. *styriaca* cultivar Gleisdorfer Rustikal), LN (CP - Lady Nail), SW (CP - Snow White), RZ (CP var. *giromontia* - Radu), SS (CP - Shine Skin), GA (CP - Greek Cultivar), GV (CP - Grey Volga), BBZ (CP var. *cylindrica* - Black Beauty), HV (CP - Hungarian Cultivar).
